# Sleep hygiene mediates anxiety and sleep quality in adults: mediation and network analysis

**DOI:** 10.1192/bjo.2026.11047

**Published:** 2026-05-11

**Authors:** Md Dilshad Manzar, Mohammed F. Salahuddin, Haitham Jahrami, Seithikurippu R. Pandi-Perumal, Ahmed S. Bahammam, Majumi M. Noohu

**Affiliations:** Department of Nursing, College of Applied Medical Sciences, Majmaah University, Saudi Arabia; Department of Pharmaceutical Sciences, School of Pharmacy, Notre Dame of Maryland University, USA; Department of Biochemistry and Molecular Genetics, University of Illinois Chicago, USA; Ministry of Health, Manama, Bahrain; Department of Psychiatry, College of Medicine and Medical Sciences, Arabian Gulf University, Bahrain; Centre for Research and Development, Chandigarh University, India; Division of Research and Development, Lovely Professional University, India; The University Sleep Disorders Center, College of Medicine, King Saud University, Saudi Arabia; National Plan for Science and Technology, College of Medicine, King Saud University, Saudi Arabia; Centre for Physiotherapy and Rehabilitation Sciences, Jamia Millia Islamia, India

**Keywords:** University students, sleep habits, sleep irregularity, sleep pattern, stress response

## Abstract

**Background:**

Sleep hygiene plays a crucial role in mental well-being, yet its influence on the relationship between anxiety and sleep quality in young adults remains underexplored.

**Aims:**

This study examines whether sleep hygiene mediates the bidirectional association between anxiety and sleep quality, identifying key behavioural targets for intervention.

**Method:**

A cross-sectional study was conducted with 405 randomly selected college students who completed validated assessments of sleep hygiene, anxiety and sleep quality. Mediation and network analyses were employed to investigate the underlying mechanisms.

**Results:**

Sleep hygiene significantly mediated the relationship between anxiety and sleep quality, emphasising the role of consistent sleep–wake schedules. Network analysis identified irregular sleep patterns and specific sleep quality components – wakefulness behaviours, sleep initiation and self-reported sleep quality – as primary factors.

**Conclusion:**

These findings highlight sleep hygiene as a modifiable, non-pharmacological strategy to mitigate anxiety and improve sleep quality. Future research should explore longitudinal interventions.

In response to the growing recognition that sleep quality has broad effects on both physical and mental health, treatment strategies have increasingly sought to include techniques for improving sleep quality. Protocols for good sleep hygiene practices are increasingly being used to manage sleep problems. Peter Hauri inaugurated this line of treatment by being the first to recommend that such sleep hygiene practices be applied to reduce insomnia symptoms.^1^ Initially, the recommended protocol consisted of adherence to a consistent bedtime at night and waking time in the morning, avoidance of caffeinated beverages and maintenance of a regular exercise routine. Since then, the original components have undergone modifications.^2^ The international classification of sleep disorders (ICSD-3) also identified inadequate sleep hygiene as one of the responses to the pressures of modern-day living, the effects of which were to reduce sleep quality and daytime wakefulness.^3^ Sleep hygiene includes practices that promote consistent sleep (e.g. regular schedules for bedtime routine, meals, activities and exercise), decrease stimulant and screen use before sleep and utilise a sleep-promoting environment (e.g. bedroom for sleep only, quiet and comfortable room).^2,3^

The attainment of optimal sleep quality is now recognised as a critical requirement for supporting overall health, and indeed represents the basis of sleep’s biological function.^4^ Phenomenologically, the achievement of high-quality sleep is experienced as a ‘satisfaction with sleep experience, integrating aspects of sleep initiation, sleep maintenance, sleep quantity, and refreshment upon waking’.^5,6^ However, it is well established that life stresses and associated anxious mental states can prevent the achievement of restful and satisfying sleep. Anxiety is one of the most common mental health issues reported by the general population, and is also widely reported among university students. It is characterised by worrying thoughts, avoidance behaviour and physical changes.^7–9^ A systematic review of reviews reported that the prevalence of anxiety was 3.8–25% in the general population and 2.0–9.1% in young adults across different cultures of the world.^10^ In one study, self-reported anxiety was found to range between 2.8 and 10.4%, with the highest prevalence among those of Euro/Anglo descent and those in Indo/Asian populations showing the lowest prevalence rates.^10^

Many studies now support the inference of a mutual dependency between sleep quality and mental and emotional health. A systematic review reported strong evidence for the bidirectionality of anxiety and sleep disturbance.^11^ Sleep and mental health are thus closely linked, with a growing number of studies showing that sleep disturbances accompany most psychiatric dysfunctionality. Whereas disturbed sleep has been seen traditionally as one of the consequences of mental health issues, a growing amount of evidence now suggests that the reverse is also true. The available data suggest that circadian dysregulation, one of the most common results of which is sleep disturbance, could potentially contribute to new mental health issues as well as the maintenance of current ones.^12–14^

The achievement of optimal sleep hygiene can significantly benefit both emotional adjustment and overall health. Conversely, this overall health status can be adversely affected by sleep disturbances. Various studies have shown that poor sleep hygiene results in decrements in both sleep quality and psychological adjustment; these associations, in turn, have feedback effects. University students, for instance, are prone to poor sleep hygiene behaviour, which reduces sleep quality and, thus, daytime alertness, cognitive resilience and problem-solving ability. These consequences can, in turn, affect self-perceptions and produce anxiety related to performance expectations. Such outcomes have a further deteriorating effect on sleep quality.^15–18^

Anxiety/stress can activate stress response that may cause sleep reactivity in vulnerable individuals. Sleep reactivity may express as cognitive–physiological arousal, predisposing individuals to insomnia and disturbed sleep. Poor sleep hygiene may increase sleep reactivity, sometimes under the influence of socioecological factors, leading to leading to poor sleep quality.^19^ Although extensive research has documented the relationships among sleep quality, anxiety and sleep hygiene individually, a significant knowledge gap exists regarding how these three factors interact in young adults without pre-existing mental health conditions. Previous studies have primarily focused on clinical populations with diagnosed mental disorders, leaving uncertainty about whether similar relationships exist in healthy young adults. Additionally, no studies have employed network analysis to identify which specific components of sleep hygiene and sleep quality drive these relationships. We hypothesised that sleep hygiene behaviour mediates the bidirectional relationship between anxiety levels and sleep quality in young adults without mental disorders. Additionally, we expected that specific dimensions of sleep hygiene and sleep quality would emerge as key drivers in this relationship network, providing potential targets for intervention strategies.

## Method

### Participants

The study was conducted between July 2023 and March 2024. The sample consisted of 405 students, representing a range of departments in a federal university (Jamia Millia Islamia, New Delhi, India), who had been selected through a simple random sampling procedure.

### Inclusion criteria

The students were initially contacted through e-mail announcements that solicited participation in the study. Five hundred students responded to the initial e-mail, all of whom were then contacted individually.

### Exclusion criteria

Students with a self-reported history of neurological disorders, chronic pain, mental illness or use of psychotropic medications were excluded. Additionally, participants with incomplete questionnaire responses were excluded from the final analysis. Following the initial screening, 450 students agreed to participate voluntarily in the study. The data of 45 students were excluded due to improper responses to scale questions or incomplete information.

We employed the sample size criteria developed by Fritz and MacKinnon for power analysis in mediation studies.^20^ The sample size (*n* = 405) of the study was adequate, based on previous simulation studies, for a power of 0.8 for medium to small sizes of path (0.39–0.14) and for the percentile bootstrap method.^20^ The local Institutional Committee for Ethics and Scientific Review of Jamia Millia Islamia (no. 19/6/450/JMI/IEC/2023) approved the study. The authors assert that all procedures contributing to this work comply with the ethical standards of the relevant national and institutional committees on human experimentation, and with the Helsinki Declaration of 1975 as revised in 2013. All procedures involving human subjects/patients were approved by the Institutional Ethics Committee of Jamia Millia Islamia.

### Design and setting

The study used a cross-sectional and observational study design, with each participant signing an informed consent form. All participants were interviewed by an experienced investigator who had close familiarity with sleep evaluation protocols. The sleep research centre at the institute routinely employs objective methods, such as polysomnography, and subjective measures of sleep evaluation. In this study sleep quality was assessed by the Leeds Sleep Evaluation Questionnaire–Mizan (LSEQ-M), and sleep hygiene behaviour by the Sleep Hygiene Index (SHI). All participants were given a summary sheet written in simple terms, describing the aims and objectives of the research. The study was further explained to them verbally until the interviewer was satisfied that they fully understood the study objectives, as well as their rights as study participants. They were also told that their participation was voluntary and risk-free and that they had the right to withdraw at any time. Additionally, it was explained that the privacy and confidentiality of all participants’ information would be strictly maintained. Various scales, written in English, were administered and followed a prescribed procedure; these assessed participants’ sleep hygiene behaviours, anxiety and sleep quality. Participants were all students at the university where the medium of instruction is English; therefore, all participants were expected to have adequate English language proficiency.

### Measures

#### Sleep hygiene behaviour

The SHI, a 13-item, self-report measure, was used to assess sleep hygiene behaviour. Quantification of subjects’ responses was based on a binary scoring system in which a ‘no’ response was coded as 0 and ‘yes’ as 1.^17^ The minimum possible score was 0, and the maximum 13;^17^ a higher score is indicative of poor sleep hygiene practice.^21^ Anwer et al recently reported that SHI had adequate psychometric validity among university students in Saudi Arabia.^17^ However, their study found that, among their university sample, self-reports of ‘poor sleep quality’ equated to a cut-off score of 7.5.^17^ In our study we used this cut-off score of 7.5 to signify the presence of poor sleep hygiene practices that may express as poor sleep symptoms.^17^ The reliability of the SHI scale in this study was acceptable, with a greatest lower bound (GLB) value of 0.70.

#### Anxiety

The Generalized Anxiety Disorder Scale (GAD-7) is a 7-item scale that was used to evaluate participants’ anxiety levels.^22^ This instrument is structured as a 7-item, self-report Likert scale for which interviewees rate their anxiety levels according to a 4-point scale (0–3) for the 2 weeks immediately preceding the test. A low score has been shown to indicate the absence of anxiety, whereas higher scores reflect the presence of bothersome anxiety. Responses to each item were summed to give the total score (0–21). The psychometric properties of GAD-7 have been well established in different populations, including university students in African–Asian countries such as Saudi Arabia and Ethiopia. Various investigations have confirmed the validity and reliability of this instrument as an anxiety measure for these populations.^23–25^ The reliability of the GAD-7 scale in this study was very good, with a GLB value of 0.885.

#### Sleep quality

LSEQ-M, which was adapted from LSEQ, was used in this study. The questionnaire is presented as a Likert scale in which respondents indicate their answer by placing a mark on a 100 mm line; the questions focus on various aspects of sleep and early morning waking behaviour. The scale was formerly used to study the effect of different intervention strategies on people with disturbed sleep, the original LSEQ having been developed to monitor sleep changes under pharmacological interventions. LSEQ-M has been found to be valid in non-pharmacological contexts, such as a sample of university students, and therefore we used the modified version in this study.^26–27^ LSEQ-M has been shown to have good psychometric properties for assessing sleep quality in collegiate students.^26–27^ Lower scores on LSEQ-M indicate poor sleep quality with insomnia symptoms.^26–27^ A cut-off score of 52.6 was shown to indicate poor sleep quality with insomnia symptoms in Ethiopian university students.^26^ The reliability of the LSEQ-M scale in this study was very good, with a GLB value of 0.821.

### Data analysis

SPSS version 26.0 (for Windows; IBM, Armonk, New York, USA; https://www.ibm.com/support/pages/downloading-ibm-spss-statistics-26-transition-extended-support-30-sep-2025) was used for carrying out all statistical analyses in this study. Participants’ characteristics are presented using descriptive statistics. The SPSS PROCESS macro for SPSS version 3.3 (for Windows; IBM, Armonk, New York, USA; https://www.ibm.com/support/pages/downloading-ibm-spss-analytic-server-330) was used to perform the mediation analysis^28^ for model-4, with the following settings: non-parametric bootstrapping with 5000 resamples and bias-corrected 95% confidence intervals, two-tailed tests and non-fixed seeding. Mediation analysis was performed using the statistical outline suggested by Zhao et al;^29^ this outline uses a slightly modified version of Baron and Kelly’s method of mediation analysis.^29,30^ Two mediation models were applied: the first evaluated the mediating effect of sleep hygiene behaviour (SHI score) on the effect of anxiety on sleep quality (LSEQ-M score). The second model evaluated the mediating effect of sleep hygiene behaviour on the effect of sleep quality on anxiety levels; the total, direct and indirect effects were then evaluated, using Preacher and Hayes’ approach to establishing the indirect effect. This approach necessitates the absence of zero from the 95% confidence interval of non-standardised coefficients.^31^

Additionally, to provide further insight into the interrelationships among sleep quality, anxiety and sleep hygiene, a network analysis was performed on the factor scores of LSEQ-M, SHI and GAD-7 using JASP 0.19.1.0 for Windows (Department of Psychological Methods, University of Amsterdam, Amsterdam, The Netherlands; https://jasp-stats.org/download/). The GAD-7 scale being unidimensional among university students,^24,25^ its total scores were used in network analysis; because SHI has three factors among university students, the three factor scores were entered into the network analysis;^32^ LSEQ-M has four factors, which were then entered into the network analysis.^26,27^ The network analysis was performed with the following settings: extended Bayesian information criteria and graphical least absolute shrinkage and selection operator estimator; autocorrelation methods; relative centrality measures; weighted networks; non-parametric bootstrap; pairwise exclusion of missing values; and a regularisation parameter (lambda) of 0.5. A moderate value of 0.5 provides a balance that usually helps avoid overcrowded insignificant edges in network analysis. In network analysis, the nodes are GAD-7 total score, four factors of LSEQ-M and factors of SHI. The edges indicate relationships between connected nodes: thick and more closely approximated edges indicate a stronger relationship. Four centrality measures – betweenness, closeness, strength and expected influence – were estimated to identify important variables in the network. All results with a *p*-value of 0.05 were considered significant.

## Results

### Participants’ characteristics

Most participating students (90.1%) were 18–25 years old, with an average age of 22.25 ± 2.84 years (Table 1). Female students comprised a majority (64.4%) of the study sample. The mean scores for GAD-7, SHI and LSEQ-M were 7.68 ± 4.94, 5.75 ± 2.15 and 61.40 ± 14.78, respectively. The prevalence of moderate–severe anxiety, poor sleep hygiene practices and poor sleep quality was 30.6, 23.7 and 29.1%, respectively (Table 1).

**Table 1:** Participants’ characteristics

Characteristics	Mean ± s.d./frequency (%)
Age, years	
Average	22.25 ± 2.84
18–25	368 (90.1)
>25	37 (9.1)
Gender	
Female	261 (64.4)
Male	144 (35.6)
GAD-7 score	7.68 ± 4.94
Anxiety symptoms^a^	
Minimal anxiety	122 (30.1)
Mild anxiety	159 (39.3)
Moderate anxiety	75 (18.5)
Severe anxiety	49 (12.1)
SHI	5.75 ± 2.15
Poor sleep hygiene^b^	
Yes	96 (23.7)
No	309 (76.3)
LSEQ-M score	61.40 ± 14.78
Poor sleep quality^c^	
Yes	118 (29.1)
No	287 (70.9)

GAD-7, Generalized Anxiety Scale-7; SHI, Sleep Hygiene Index; LSEQ-M, Leeds Sleep Evaluation Questionnaire–Mizan.

a. Based on GAD-7 score.

b. Based on SHI score.

c. Based on LSEQ-M score.

### Sleep hygiene behaviour mediates the effect of anxiety on sleep quality

Sleep hygiene behaviour was a significant mediator in the relationship between anxiety and sleep quality (Table 2 and Supplementary Fig. 1), accounting for about 27.15% of variance in sleep quality (*R* ^2^ = 0.2715, *F*[1, 402] = 0.74.91, *p* < 0.001). Anxiety was associated with sleep hygiene (*b* = 0.20, s.e. = 0.02, *p* < 0.001), implying that anxious participants were more likely to have poor sleep hygiene habits. Sleep hygiene was also associated with sleep quality (*b* = −1.88, s.e. = 0.33, *p* < 0.001): i.e. participants with poor sleep hygiene behaviour were more likely to have poor sleep quality. Higher anxiety levels were associated with poor sleep quality after adjusting for sleep hygiene behaviour (*b* = −1.00, s.e. = 0.14, *p* < 0.001). The indirect effect of the mediation model was significant, as determined by the absence of zero in the bootstrapped 95% confidence interval (−0.38 (−0.51, −0.25)).

**Table 2:** Mediating role of sleep hygiene behaviour on anxiety and sleep quality

Independent variable	Outcome variable	*β*	*b*	s.e.	95% bootstrapping CI		*P*-value
					LL	UL	
Anxiety	Sleep hygiene practices	0.46	0.20	0.02	0.16	0.24	<0.001
Sleep hygiene practices	Sleep quality	−0.27	−1.88	0.33	−2.53	−1.23	<0.001
Anxiety (direct effect)	Sleep quality	−0.34	−1.00	0.14	−1.29	−0.72	<0.001

Types of effect	*b*	s.e.	95% bootstrapping CI		*P*-value
			LL	UL	
Total effect	−1.38	0.13	−1.64	−1.12	<0.001
Indirect effect	−0.38	0.07	−0.51	−0.25	–

*β,* standardised coefficient; *b*, unstandardised coefficient; LL, lower limit; UL, upper limit.

Sleep hygiene practices, anxiety and sleep quality were assessed by the Sleep Hygiene Index, Generalized Anxiety Scale-7 and Leeds Sleep Evaluation Questionnaire−Mizan, respectively.

### Reverse model: sleep hygiene mediates the effect of sleep quality on anxiety

Sleep hygiene behaviour was a significant mediator in the relationship between sleep quality and anxiety level (Table 3 and Supplementary Fig. 2), accounting for about 29.59% of the variance in sleep quality (*R* ^2^ = 0.2959, *F*[1, 402] = 0.84.49, *p* < 0.001). Sleep quality was associated with sleep hygiene (*b* = −0.06, s.e. = 0.01, *p* < 0.001), suggesting that participants with poor sleep quality were also likely to have poor sleep hygiene behaviour. Poor sleep hygiene was associated with higher anxiety levels (*b* = 0.73, s.e. = 0.11, *p* < 0.001). The direct effect of sleep quality on anxiety level was significant (*b* = −0.11, s.e. = 0.02, *p* < 0.001) after adjusting for sleep hygiene behaviour. The indirect effect of the reverse mediation model was significant, as determined by the absence of zero in the bootstrapped 95% confidence interval (−0.04 (−0.06, −0.03)).

**Table 3:** Mediating role of sleep hygiene behaviour on sleep quality and anxiety

Independent variable	Outcome variable	*β*	*b*	s.e.	95% bootstrapping CI		*P*-value
					LL	UL	
Sleep quality	Sleep hygiene practices	−0.43	−0.06	0.01	−0.08	−0.05	<0.001
Sleep hygiene practices	Anxiety	0.32	0.73	0.11	0.52	0.94	<0.001
Sleep quality (direct effect)	Anxiety	−0.32	−0.11	0.02	−0.14	−0.08	<0.001

Types of effect	*b*	s.e.	95% bootstrapping CI		*P*-value
			LL	UL	
Total effect	−0.15	0.01	−0.18	−0.12	<0.001
Indirect effect	−0.04	0.01	−0.06	−0.03	–

*β,* standardised coefficient; *b*, unstandardised coefficient; LL, lower limit; UL, upper limit.

Sleep hygiene practices, anxiety and sleep quality were assessed by the Sleep Hygiene Index, Generalized Anxiety Scale-7 and Leeds Sleep Evaluation Questionnaire−Mizan, respectively.

### Network analysis

**Fig. 1:**
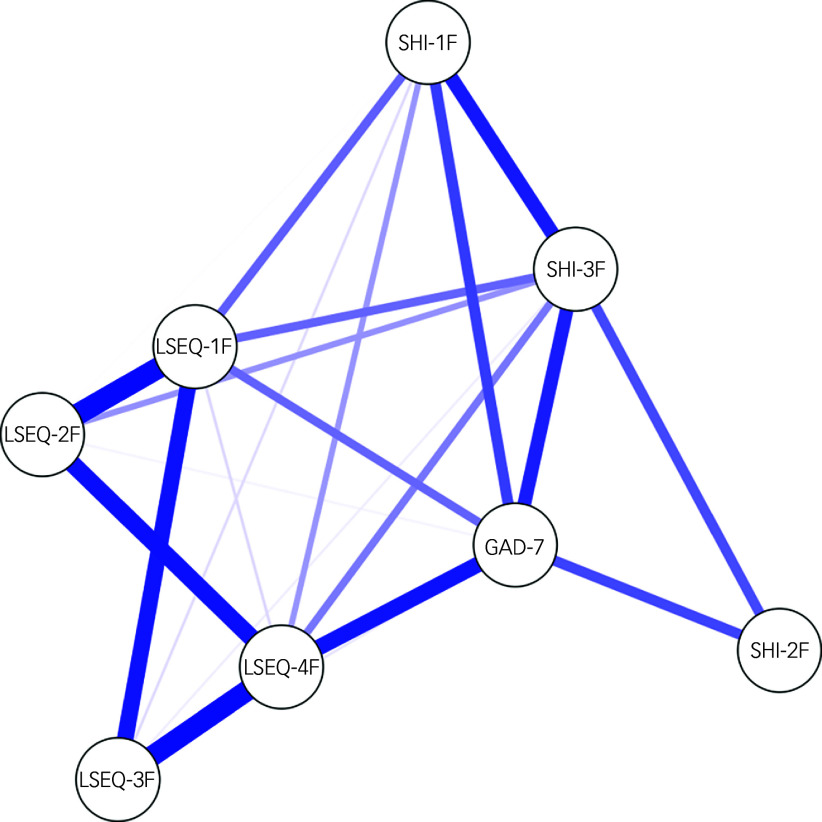
The network plot of relationships among factor scores of sleep hygiene behaviour (SHI score), sleep quality (LSEQ-M score) and anxiety level (GAD-7 score). SHI, Sleep Hygiene Index; LSEQ-M, Leeds Sleep Evaluation Questionnaire–Mizan version; GAD-7, Generalized Anxiety Scale-7. LSEQ-1F, LSEQ-2F, LSEQ-3F and LSEQ-4F are factor scores of LSEQ; SHI-1F, SHI-2F and SHI-3F are factor scores of SHI.

The network analysis was used to identify factors/dimensions of LSEQ-M, SHI and the GAD-7 scale that had an important bearing on the interrelationships of sleep quality, anxiety and sleep hygiene (Fig. 1). The factor scores of LSEQ-M are LSEQ-1F (getting to bed), LSEQ-2F (quality of sleep), LSEQ-3F (awake following sleep) and LSEQ-4F (behaviour following waking). The factor scores of SHI are SHI-1F (BRB: bed-related behaviour), SHI-2F (BRC-A: bed-related cognitive activity) and SHI-3F (SWT: sleep–wake time). LSEQ-M factor scores shared strong inter-factor correlations. Similarly, SHI factor scores shared strong inter-factor correlations but these were relatively weaker than those between LSEQ-M factors (Fig. 1). All three factors of SHI shared direct and indirect connections with GAD-7 score; two of these factors, i.e. sleep–wake time and bed-related behaviour, also shared direct, but relatively weaker, connections with two factors of LSEQ-M, behaviour following waking and getting to bed. Two factors of LSEQ-M shared strong direct connections with GAD-7 score: behaviour following waking and GAD-7, whereas getting to bed and GAD-7 were relatively weak.

The network analysis was employed to determine centrality indices for identification of key factors in the network model (Table 4). For betweenness centrality – a measure of the degree of connectivity – the GAD-7 total score had the highest values, followed by LSEQ-4F, LSEQ-1F, LSEQ-2F and SHI-3F. For closeness centrality – a measure of the shortest average distance to all other nodes – the GAD-7 total score had the highest values, followed by LSEQ-4F, LSEQ-1F, SHI-3F and LSEQ-2F. For strength centrality – a measure of weighted strong connections – LSEQ-4F had the highest values, followed by LSEQ-1F, GAD-7 total score, SHI-3F and LSEQ-2F. Finally, for expected influence centrality – a measure predicting influence by acting as a bridge between adjacent nodes – LSEQ-4F had the highest values, followed by LSEQ-1F, GAD-7 total score, SHI-3F and LSEQ-2F.

**Table 4:** Centrality measures of factor scores for sleep quality (LSEQ-M), sleep hygiene (SHI) and anxiety (GAD-7) among university-attending young adults with no history of mental disorder

Factor scores of LSEQ-M, SHI and GAD-7	Betweenness	Closeness	Strength	Expected influence
LSEQ-1F	0.429	0.853	0.940	0.940
LSEQ-4F	0.857	0.943	1.000	1.000
GAD-7	1.000	1.000	0.881	0.881
LSEQ-2F	0.143	0.775	0.666	0.666
LSEQ-3F	0	0.762	0.609	0.609
SHI-1F	0	0.712	0.589	0.589
SHI-2F	0	0.576	0.289	0.289
SHI-3F	0.143	0.794	0.862	0.862

LSEQ-M, Leeds Sleep Evaluation Questionnaire−Mizan; SHI, Sleep Hygiene Index; GAD-7, Generalized Anxiety Scale-7.

LSEQ-1F, LSEQ-2F, LSEQ-3F and LSEQ-4F are factor scores of LSEQ; SHI-1F, SHI-2F and SHI-3F are factor scores of SHI.

## Discussion

To the best of our knowledge, this is the first study to establish the sleep hygiene–anxiety–sleep quality interrelationship, as well as to identify the specific dimensions of sleep hygiene and sleep quality that drive this interrelationship network in a sample of collegiate young adults without a history of mental disorders. It was found that sleep hygiene behaviour significantly mediates the bidirectional relationship between anxiety and sleep quality. The findings of the network analysis supplemented the outcome of the mediation analysis. The important factors/dimensions of the interrelationships among sleep hygiene, severity of anxiety symptoms and sleep quality were identified. Out of 13 questions measuring sleep hygiene behaviour, only 2 measuring behaviour of going to bed at different times and getting out of bed at different times, both part of SHI-3F, were major contributors to the bidirectional relationship between poor sleep quality and increasing severity of anxiety. Furthermore, SHI-1 (bed-related behaviour) also contributed partly to the bidirectional relationship between poor sleep quality and increasing severity of anxiety. With respect to the dimension of sleep quality associated with increasing severity of anxiety symptoms, behaviour following wakefulness (LSEQ-4F) was most affected, followed by getting to sleep (LSEQ-1F) and self-reported sleep quality (LSEQ-2F). The novel findings about specific features of sleep hygiene, and sleep quality associated with increasing severity of anxiety symptoms, among collegiate young adults will help in developing targeted interventions. The findings of this study provide specific dimensions of sleep hygiene and sleep quality that may be targeted by modification strategies for the management of sleep-related anxiety symptoms in young adults with no history of mental disorders.

These findings have direct clinical applications for university health services. Healthcare providers should implement dual-targeted interventions that address both sleep hygiene and anxiety symptoms. Specifically, counsellors should focus on methods to regularise the sleep–wake schedules and morning routines of students, because these emerged as the strongest mediating factors. Simple interventions such as sleep-scheduling apps or morning routine checklists could be effective first-line treatments before considering pharmacological approaches. Additional prevention and management strategies may explore the bed-related behaviour factor of sleep hygiene: stress management before bedtime and socioecological aspects, such as an uncomfortable bed, bedroom and screen and stimulant use before bedtime, etc. University students might benefit from the implementation of a holistic programme based on nature quotient for managing and mastering their socioecological resources in a sleep hygiene promotion.^33^

The bidirectional association between anxiety and chronic stress with underlying neurobiological mechanisms is well established.^34–36^ Consistent with these findings, we previously reported that the relationship between stress and anxiety is mediated by insomnia.^25^ However, the role of sleep hygiene behaviour in mediating the relationship between anxiety and sleep quality has not been studied extensively in young adults with no medical history of mental disorders. The mediation analysis in the present study showed that sleep hygiene behaviour mediates the bidirectional relationship between anxiety and sleep quality. Various studies have reported that anxiety is associated with sleep quality.^37,38^ Furthermore, the bidirectional association between sleep and anxiety has also been reported.^39^ Our findings agree with those of previously reported studies, and we have additionally identified and confirmed a novel concept that sleep hygiene mediates the effect of sleep quality on anxiety, and vice versa. Furthermore, the neuroanatomical basis of the link between psychological stress and sleep quality has been explored in a recent study, in which data from 318 healthy students were collected using the Pittsburgh Sleep Quality Index, the Psychosomatic Tension Relaxation Inventory and voxel-based morphometry; in that study, psychological stress was found to have a detrimental effect on sleep quality. According to mediation analysis results, the association between psychological stress and sleep quality was partially mediated by the region of the bilateral inferior temporal gyrus.^40^

Certain areas of the brain, including the hypothalamus, amygdala, prefrontal cortex and brainstem nuclei, were found to be activated during anxiety and also to have a role in regulating the sleep–wake cycle.^41,42^ Neurobiological evidence also favours the view that anxiety levels can influence sleep quality.^40^ The intricate connections in the central nervous system may account for the inverse relationship between sleep quality and anxiety level. Anxiety and depression comorbidity status was found to affect the severity of insomnia; moreover, this relationship was mediated by poor sleep hygiene behaviour.^43^ The bidirectional relationship between anxiety and sleep quality can be explained through a cyclical model. Poor sleep hygiene disrupts the natural sleep–wake rhythm, leading to increased activation of the hypothalamic–pituitary–adrenal axis. This heightened physiological arousal manifests as anxiety symptoms which, in turn, make it harder to maintain consistent sleep patterns. The mediation effect of sleep hygiene suggests that it acts as a crucial breaking point in this cycle, where improved sleep habits can potentially interrupt both pathways of this bidirectional relationship.

Taken together, these findings suggest that anxiety is closely associated with, and is detrimental to, the sleep quality that is experienced. The study findings also suggest that anxiety reduction should be a key focus in intervention strategies available for college students, since this would facilitate the development of positive sleep hygiene behaviour.

According to another study from Japan, cognitive assessments influenced the connection between sleep habits and associated stress responses. The findings also showed that maintaining a moderate commitment to treatment goals may be critical for lowering anxiety, particularly given the difficulties in changing long-established behaviours. The outcomes of that study should be relevant to lifestyle-related health education.^44^

Future research should focus on three specific areas: (a) longitudinal studies tracking the temporal relationship between sleep hygiene improvement and anxiety reduction; (b) randomised controlled trials comparing different sleep hygiene interventions targeting the specific factors identified in our network analysis; and (c) investigation of potential moderating variables, such as academic stress levels, that may depend on academic department and session of enrolment, chronotype and social media use patterns. Additionally, studies using objective sleep measures such as actigraphy, alongside subjective assessments, would help validate these relationships. The methods used to investigate the mediating role of sleep hygiene practices possess limitations that accrue from a cross-sectional study and, ideally, a longitudinal design would have been preferable. Another potential limitation is that the scale used to assess anxiety measured this factor for only 2 weeks preceding testing time. Additionally, there were no objective measures used to assess the variables recorded in the study. Therefore, it is recommended that further confirmatory studies using such measures be carried out, recording potential confounding due to examination, and that these studies should employ sample sizes larger than those used in the current study. Persons with anxiety symptoms often have depressive features. Therefore, future studies should explore key depressive symptoms and include these in the network analysis for a comprehensive clinical understanding of the relationships among sleep quality, sleep hygiene and anxiety.

### Implications in practice

This study has important implications for university students, university mental health centres and medical practitioners. By integrating a structured routine of the sleep–wake cycle, medical practitioners can mitigate sleep-related anxiety in collegiate adults.

In conclusion, sleep hygiene behaviour is an important factor that influences the bidirectional relationship between anxiety and sleep among university-attending young adults with no history of mental disorders. The major aspects of sleep hygiene (i.e. varying bedtimes and waking times) and sleep quality (i.e. behaviours following wakefulness, getting to sleep and self-reported sleep quality) driving this bidirectional relationship may be targeted by counselling intervention strategies to manage health outcomes among young adults.

## Supporting information

10.1192/bjo.2026.11047.sm001 Manzar et al. supplementary material 1 Manzar et al. supplementary material

10.1192/bjo.2026.11047.sm002 Manzar et al. supplementary material 2 Manzar et al. supplementary material

## Data Availability

The data that support the findings of this study are available from the corresponding author, M.F.S., upon reasonable request.

## Supplementary material

The supplementary material is available online at https://doi.org/10.1192/bjo.2026.11047
