# Supplementary material for: Sleep hygiene mediates anxiety and sleep quality in adults: mediation and network analysis
Source: BJPsych Open. 2026 May 11;12(3):e134. doi: 10.1192/bjo.2026.11047 (PMC13169052; doi:10.1192/bjo.2026.11047)
Supplement: Manzar et al. supplementary material 2 — Manzar et al. supplementary material [file S2056472426110473sup002.docx]

# Sleep quality

Anxiety

## Sleep Hygiene behavior

**-.06 (.01)***

**.73 (.11)***

**-.11 (.02)*, -.04^a^**

**Figure** 2. The model with sleep hygiene behavior (SHI score) as a mediator in the effect of sleep quality (LSEQ-M score) on anxiety level (GAD-7 score).

Note. The first values are the unstandardized coefficients, second values under brackets are standard errors; **p<.001; and* ^a^overall indirect effect. The indirect effect of sleep quality (LSEQ-M score) on anxiety level (GAD-7 score) through sleep hygiene behavior (SHI score) was significant (95% confidence interval -.04 [-.06, -.03]).

Sleep hygiene behavior, anxiety, sleep quality were assessed by the Sleep Hygiene Index (SHI), Generalized anxiety scale-7 (GAD-7), and Leeds sleep evaluation questionnaire-Mizan version (LSEQ-M), respectively.
